# Diversity of respiratory viruses present in nasal swabs under influenza suspicion in respiratory disease cases of weaned pigs

**DOI:** 10.3389/fvets.2022.1014475

**Published:** 2022-10-19

**Authors:** Gerard E. Martín-Valls, Yanli Li, Ivan Díaz, Esmeralda Cano, Silvana Sosa-Portugal, Enric Mateu

**Affiliations:** ^1^Department de Sanitat i Anatomia Animals, Faculty of Veterinària, Universitat Autònoma de Barcelona, Cerdanyola del Vallès, Spain; ^2^IRTA, Programa de Sanitat Animal, Centre de Recerca en Sanitat Animal (CReSA), Campus de la Universitat Autònoma de Barcelona (UAB), Barcelona, Spain

**Keywords:** respiratory viruses, pig nursery, *influenza A*, respiratory disease porcine respiratory disease complex, porcine reproductive and respiratory syndrome virus, swine orthopneumovirus, porcine cytomegalovirus

## Abstract

Respiratory diseases in weaned pigs are a common problem, with a complex etiology involving both viruses and bacteria. In the present study, we investigated the presence of eleven viruses in nasal swabs, collected from nurseries (55 cases) under the suspicion of *swine influenza A virus* (swIAV) and submitted by swine veterinarians for diagnosis. The other ten viruses included in the study were influenza B (IBV) and D (IDV), *Porcine reproductive and respiratory syndrome virus* (PRRSV), *Porcine respiratory coronavirus* (PRCV), *Porcine cytomegalovirus* (PCMV), *Porcine circovirus* 2 (PCV2), 3 (PCV3) and 4 (PCV), *Porcine parainfluenza 1* (PPIV1) and *Swine orthopneumovirus* (SOV). Twenty-six swIAV-positive cases and twenty-nine cases of swIAV-negative respiratory disease were primarily established. While IBV, IDV, PCV4 and PPIV1 were not found in any of the cases, PRCV, SOV, and PCMV were more likely to be found in swIAV-positive nurseries with respiratory disease (*p* < 0.05). Overall, PCV3, PRRSV, and PCMV were the most frequently detected agents at herd level. Taken individually, virus prevalence was: swIAV, 48.6%; PRCV, 48.0%; PRRSV, 31.6%; SOV, 33.8%; PCMV, 48.3%, PCV2, 36.0%; and PCV3, 33.0%. Moreover, low Ct values (<30) were common for all agents, except PCV2 and PCV3. When the correlation between pathogens was individually examined, the presence of PRRSV was negatively correlated with swIAV and PRCV, while was positively associated to PCMV (*p* < 0.05). Also, PRCV and SOV were positively correlated between them and negatively with PCMV. Besides, the analysis of suckling pig samples, collected in subclinically infected farrowing units under an influenza monitoring program, showed that circulation of PRCV, PCMV, SOV, and PCV3 started during the early weeks of life. Interestingly, in those subclinically infected units, none of the pathogens was found to be correlated to any other. Overall, our data may contribute to a better understanding of the complex etiology and epidemiology of respiratory diseases in weaners. This is the first report of SOV in Spain and shows, for the first time, the dynamics of this pathogen in swine farms.

## Highlights

- Thirty-one virus combinations suggest a complex etiology of pig respiratory diseases.
- First detection and dynamics description of SOV in Spanish pig farms.
- PRRSV is negatively correlated with swIAV and PRCV, while positively with PCMV.
- SOV, PCMV, PRCV and PCV3 detection at early ages suggests an important role of sows.

## Introduction

Respiratory diseases are one of the most common problems in weaned and growing pigs, with a complex etiology involving both viral and bacterial agents (1–4). The most common clinical picture is characterized by cough, with or without fever and possibly nasal discharge, labored breathing, and increased mortality. Since several agents may produce similar lesions, the pathological picture has an indicative value, but does not allow precise diagnosis. In many nurseries, the outbreaks are recurrent batch after batch (5).

Agents causing respiratory diseases are often categorized as primary and secondary/opportunistic pathogens. Primary respiratory infectious agents are capable of subverting host respiratory defense barriers and establish infections on their own, while opportunistic agents take advantage of the damage caused by primary agents to establish the infections. Primary infections are often complicated by opportunistic agents, resulting in more serious respiratory disease outcomes. Some agents could act both as primary and opportunistic invaders, depending on environmental conditions and the homeostasis of pigs. Viral pathogens are generally considered as primary agents of respiratory disease in nurseries. In the field, it is common to detect several respiratory viruses simultaneously circulating in nurseries, making difficult to ascertain the role of each one, or their interactions in a particular case. For some pathogens like swine *influenza A virus* (swIAV), a primary role can be assumed despite the variation of virulence among strains (1, 6). For others, like *porcine respiratory coronavirus* (PRCV), their role in respiratory disorders is difficult to be established (7, 8). Additionally, agents, such as *porcine circovirus 2* (PCV2) and *porcine reproductive and respiratory syndrome virus* (PRRSV), may impair or modify host defense mechanisms against other agents (9–13). The immunosuppressive features of *porcine cytomegalovirus* (PCMV) have not been fully proved, although it is supposed to regulate the expression of different cytokines (IL-1, IL-2, IL-12, TNF-α, and IL-10) (14, 15).

Bacterial agents also participate in the respiratory disease of weaners. Some, *i.e., Mycoplasma hyopneumoniae*, are capable of causing primary respiratory disease, while others may mainly act as opportunistic agents. The interactions leading to such complications are poorly understood, the available evidence are partial and sometimes contradictory (16–18).

Nevertheless, a consensus view from the clinical and pathological picture points to the interaction of different agents with host genetic background and its immune status, under specific environmental circumstances [see Saade (19) for an excellent review].

In recent years, several viruses have been added to the list of potential porcine respiratory pathogens. Among them, it is worth mentioning Influenza B (IBV) and D (IDV) (20–23), *swine orthopneumovirus* (SOV) (24), *porcine parainfluenza 1 virus* (PPIV1, also designated as *porcine respirovirus*) (25–27), and *porcine circovirus 3* (PCV3) (28). The knowledge on the epidemiology of these viruses, their interactions and participation in respiratory disease is limited, with only a few reports available (29–33). The role of other recently discovered agents, such as PCV4 (34) is not known yet (35).

The present study aimed to explore the frequency and combinations of several respiratory viruses, including SOV, PPIV, PCV3 and PCV4, together with IBV and IDV, in nasal swabs collected from pigs in respiratory disease outbreaks. Swabs were submitted to the laboratory by swine veterinarians under suspicion of swIAV infection.

## Materials and methods

### Cases and sample collection

The study comprised 55 cases, corresponding to the number of respiratory disease outbreaks in nurseries (out of the 84 received for diagnosis between 2017 and 2019) from Spain and Portugal. Nasal swabs (*n* = 873) were submitted for diagnosis under suspicion of swIAV. Twenty-nine cases were discarded when <10 animals were sampled or did not fulfill any of the criteria: (1) Noticeable respiratory disease was reported in nurseries, (2) cough and fever were predominant signs in diseased animals, with mortality association above-usual in the farm, and (3) nasal swabs were stored at 4°C and submitted to the laboratory (*Laboratori Veterinari de Diagnosi en Malalties Infeccioses*, UAB) in <24 h after collection.

All cases were initially examined for swIAV presence during a swIAV surveillance project carried out in Spanish farms (36). The 55 examined cases comprised both swIAV-positive (*N* = 26), as well as swIAV-negative (*N* = 29). Nasal swab suspensions of each submission were pooled (3–4 samples/pool) for the initial analysis. Then, for each pathogen present, 10 corresponding positive farms were randomly selected from positive cases, and animals were analyzed individually. Each farm was assigned a code for the purpose of anonymized identification.

Additionally, since some other farms performed routine monitoring of swIAV in farrowing crates and nurseries, we had the opportunity to test the circulation of respiratory viruses at different ages in 8 additional farms, where animals did not show overt respiratory disease. This sampling comprised 20 nasal swabs collected from farrowing units and 12 from nurseries. With those numbers, any agent could be detected if present in ≥15 or ≥25% of the animals in the farrowing units and nurseries, respectively, (95% confidence). These samples were initially examined by pooling (2–3 samples/pool).

### Swab processing, nucleic acid extraction and RT-PCR

Each nasal swab tip (Sigma-Virocult) was submerged into 1 ml viral transport medium immediately after collection. Upon reception, swabs were resuspended in the transport medium, tubes were vortexed, and three aliquots were stored at −80°C until needed. Before nucleic acid extraction, thawed aliquots were centrifuged at 8,000 g during 5 min at 4°C to collect supernatants for RNA/DNA extraction (MagMax Core nucleic acid extraction kit, ThermoFisher Scientific). All analyses were carried out with the same RNA batch (a single extraction per sample). Exogenous internal positive control (IPC) from PRRSV LSI Vetmax PRRS EU/NA kit (Life technologies) was used to validate extraction proficiency. Samples known to be swIAV-positive were also included as positive control for nucleic acid extraction.

The presence of different viruses was examined by either real-time PCR or real-time RT-PCR using AgPath-ID One-Step RT-PCR Reagents (ThermoFisher Scientific). SwIAV, IBV and IDV, and PCV2, PCV3, and PCV4 were detected using previously described primers (37–42). For PPV1, PCMV, PRCV, and SOV, the primers were specifically designed (see Table 1). All PCRs were performed using 5 μl of the extracted nucleic acids, except for PRRSV, where 7 μl was used, as instructed by LSI VetMax PRRS EU/NA v2 (Life Technologies). The primer specificity was evaluated by SANGER sequencing of the amplified products of samples positive by conventional RT-PCR (see primers in Table 1). In each round of PCR, positive (samples containing the target virus as confirmed by sequencing) and negative controls (PBS) were included.

**Table 1:** List of primers and probes designed for the present study.

**Primer**	**Sequence**	**Ref. accession**
**Real time PCR**
*PPV-1 Fw*	ATTCGGGCAGAGATGTAACG	MF681710
*PPV-1 Rv*	TATCCCACCCCTCGGTCTAT	
*PPV-1 Probe*	FAM-GCCCGATAAAATCCACAAAGA-BHQ1	
*SOV Fw*	GGGGAGGACTTGATGCTGTA	KX364383.1
*SOV Rv*	AACTTTGCTGCCTCCTTTGA	
*SOV Probe*	FAM-CTGAAAGCTGAGAAGGCCAG-BHQ1	
*PCMV Fw*	AATGCGTTTTACAACTTCACG	KF017583.1
*PCMV Rv*	CTGAGCATGTCCCGCCCTAT	
*PCMV Probe*	FAM-CTCTAGCGGCGTCCATCACC-BHQ1	
*PRCV Fw*	TCAGCCAATTTTGGTGACAG	KY406735.1
*PRCV Rv*	GATCATCCTTTGGCAAGTGG	
*PRCV Probe*	FAM-ATGGGAGCAGTGCTAAGCAT-BHQ1	
**End-point PCR**
*PRCV Fw*	AAACACTACTTGTGGTTTTGGTTAT	MF462726
*PRCV Rv*	ACAGTCACACCGAACGGAAT	
*PCMV Fw*	TGACAGTGAGCAGTCGGAAT	KX575707.1
*PCMV Rv*	TCAGGCGTGGATATGTAACG	
*SOV Fw*	CTATCGGAACCGAATGAGAC	KX364383.1
*SOV Rv*	TGCCAGGAGCCATATTTG	

To evaluate farm prevalence with more precision, individual samples from 10 cases positive for each pathogen were also analyzed by RT-PCR.

### Statistical analysis

A farm was considered infected by a given virus if at least one sample was positive for that virus. Thus, for each virus, its presence or absence in the farm was coded as 1/0 or Yes/No depending on the purpose of the analysis. The associations between pathogens at a farm level were initially tested using a tetrachoric correlation matrix (tetrachoric function in R).

An Exploratory Factor Analysis was then performed to identify the underlying relationships between different viruses (packages RcmdrMisc and psych in R). The Kaiser-Meyer-Olkin (KMO) test was performed to determine sampling adequacy and the Bartlett's test to determine sphericity. Eigenvalues were used to determine the number of factors to extract (eigenvalues ≥ 1.0). Factor loadings were calculated to identify whether a variable was not loaded sufficiently onto any given factor (loading <0.40).

The final step was to elaborate regression models, where one virus (presence or absence) was considered the respondent variable and the others as independent factors (lm function in R). When two or more variables were found to be correlated in the correlation analysis, the interactions between variables were included in the formula. The Breusch-Pagan test was used to test the heteroscedasticity, whereby regression was weighted. The variance inflation factor was calculated to determine how much collinearity existed in the model. The same scheme of data analysis was applied to the examination of the database composed of individual results.

Individual results from 10 outbreaks were used to determine the prevalence of these viruses in respiratory outbreaks. In this case, the average and distribution of Ct-values were also compared using Kruskal-Wallis test. All analyses were done in RStudio 2021.09.2 + 382 “Ghost Orchid” Release for Windows.

## Results

### Prevalence and association of respiratory viruses PRRSV, PRCV, SoV, PCV2, PCV3, and PCMV in swIAV-positive and -negative nurseries

The final sampling comprised 55 respiratory disease cases, 26 of which in swIAV-positive nurseries. Overall, PCV3 was the most frequently detected agent, present in 43/55 cases (78.2%; CI_95%_: 66.6–87.8%). PRRSV and PCMV were found in 40/55 cases (72.7%; CI_95%_: 58.8–83.5%), PRCV in 29/55 cases (52.7%; CI_95%_: 39.3–66.1%), PCV2 in 18/55 cases (32.7%; CI_95%_: 21.1–46.8%), and SOV in 17/55 cases (30.9%; CI_95%_: 19.5–45.0). IBV, IDV, PCV4, and PPIV1 were not detected in any case. To note, this is the first description of SOV occurrence in pig herds in Spain and the second in Europe.

**Figure 1:**
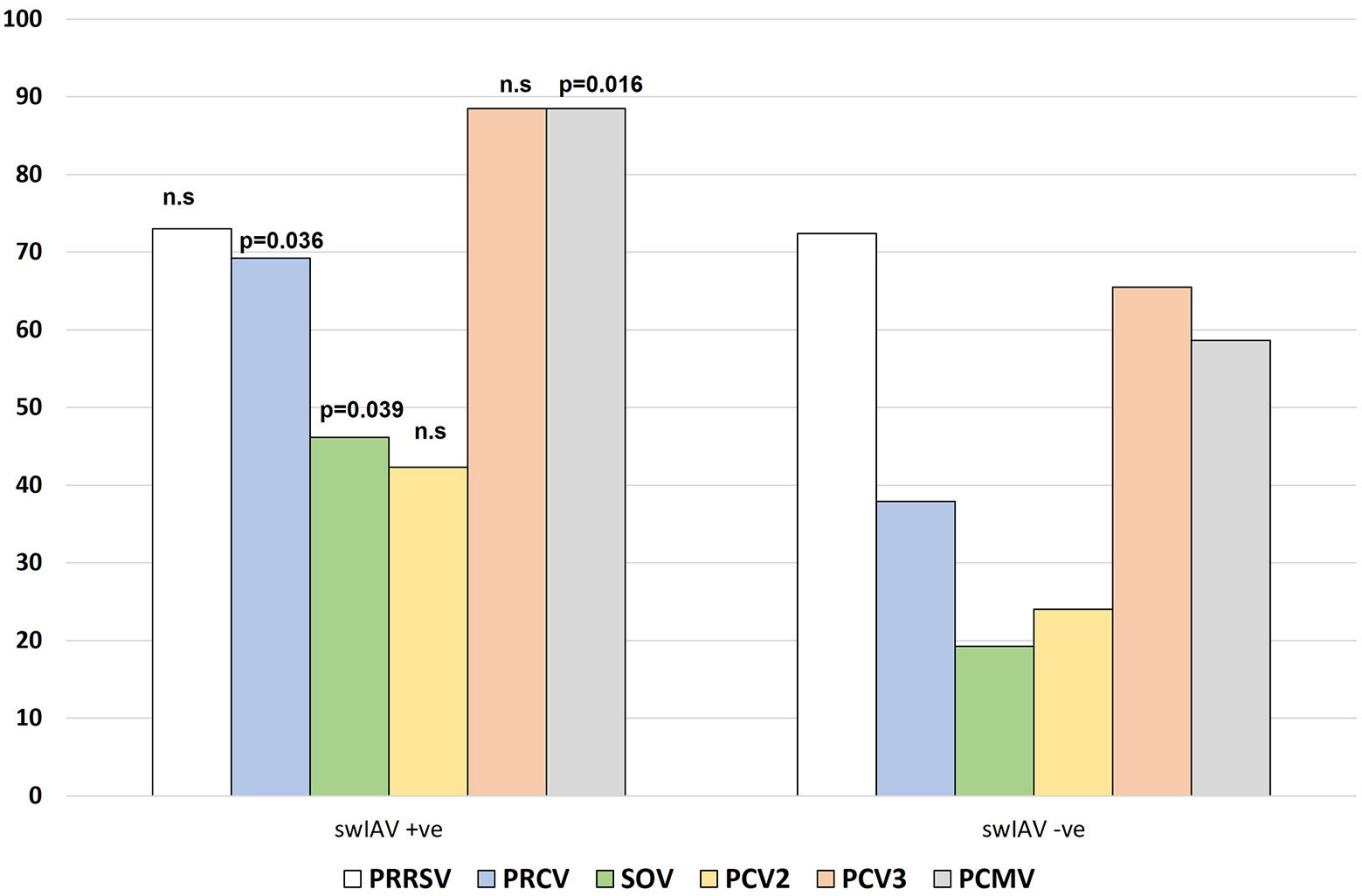
Frequency of detection of the different viruses in the studied cases, based on the swIAV status of the nursery. n.s, not significative.

Figure 1 shows the proportion of cases in which each pathogen (PRRSV, PRCV, SOV, PCV2, PCV3, PCMV) was found based on swIAV status of the farm. Interestingly, the frequency of a given pathogen detected in swIAV-positive cases (on a herd level) varied from that in swIAV-negative cases. PRCV, SOV, and PCMV were more likely to be found in swIAV-positive nurseries (69.2 vs. 37.9% for PRCV; 46.2 vs. 19.2% for SOV and 88.5 vs. 58.6% for PCMV, *p* < 0.05). Among 55 farms, 31 virus combination patterns were displayed (Table 2). In one respiratory outbreak, none of the examined viruses was detected.

**Table 2:** Distribution of the 55 examined farms according to the combination of respiratory viruses found in the nurseries.

**swIAV**	**PRRSV**	**PRCV**	**SOV**	**PCV2**	**PCV3**	**PCVM**	**N°farms**
-	-	-	-	-	-	-	1
-	-	-	-	-	-	+	1
-	-	-	-	-	+	-	3
-	-	-	-	+	+	-	1
-	+	-	-	-	-	-	2
-	+	-	-	-	+	-	3
-	+	-	-	-	-	+	2
-	+	-	+	-	-	+	1
-	+	-	-	-	+	+	1
-	+	-	-	+	-	+	1
-	+	-	-	+	+	+	2
-	+	+	-	-	+	-	1
-	+	+	-	+	+	-	1
-	+	+	-	-	+	+	2
-	+	+	+	-	+	+	3
-	+	+	-	+	+	+	2
+	-	-	+	-	+	+	1
+	-	-	-	+	+	+	1
+	-	+	-	-	+	+	3
+	-	+	+	+	+	+	2
+	+	-	-	-	-	-	1
+	+	-	-	-	+	-	2
+	+	-	-	+	+	-	1
+	+	-	-	-	+	+	2
+	+	-	-	+	+	+	1
+	+	+	+	-	-	+	1
+	+	+	-	-	+	+	3
+	+	+	+	-	+	+	3
+	+	+	+	+	-	+	1
+	+	+	-	+	+	+	1
+	+	+	+	+	+	+	4

None of the farms were positive for IBV, IDV, PCV4 or PPIV1.

Next, a tetrachoric correlation matrix was calculated to determine associations between different pathogens at a farm level. The results showed that the swIAV-positive status was positively correlated with the presence of PRCV, SOV, and PCMV (*p* < 0.05) (Table 3). The presence of PRCV was correlated with PCV3 (*p* < 0.05) SOV, and PCMV (*p* < 0.001).

**Table 3:** Tetrachoric correlation coefficients.

	**swIAV**	**PRRSV**	**PRCV**	**SOV**	**PCV2**	**PCV3**	**PCMV**
**Farm level**
swIAV	1.00						
PRRSV	−0.07	1.00					
PRCV	0.50*	0.22	1.00				
SOV	0.52*	0.15	0.75***	1.00			
PCV2	0.25	0.05	0.25	0.24	1.00		
PCV3	0.41	−0.14	0.57*	0.05	0.32	1.00	
PCMV	0.52*	0.19	0.75***	0.75**	0.36	0.14	1.00
**Individual level**
swIAV	1.00						
PRRSV	−0.59*	1.00					
PRCV	0.14	−0.63*	1.00				
SOV	0.31	−0.29	0.71***	1.00			
PCV2	0.40	−0.05	0.13	0.69**	1.00		
PCV3	−0.27	0.04	−0.30	−0.31	−0.12	1.00	
PCMV	−0.32	0.52^†^	−0.82***	−0.84	−0.28	0.44	1.00

The table shows the results of the tetrachoric correlation matrices for the presence or absence of each virus at farm level (upper) or in individuals (lower). ^†^p = 0.06; *p < 0.05; **p < 0.01; ***p < 0.001.

The exploratory factor analysis with two factors explained 40.2% of the total variance. Factor 1 included all viruses but PCV3 and Factor 2 included swIAV, SOV, PCV2, and PCV3. Detailed results are shown in Supplementary material 1.

The regression analysis at the farm level showed a significant model only for PRCV. The presence of PRCV in the farm was significantly (*p* < 0.05) related to the presence of SOV, PCV3, and probably PCMV (*p* = 0.051) (statistics shown in Table 4A).

**Table 4:** Regression models. The tables show the significant regression models at a farm level (A) or individual level (B).

**A. Farm level**				
Formula = PRCV ~ swIAV + PRRSV + PRRSV *swIAV + SOV + PCMV + PCV3 + PCV2 + PRCV*PCMV *SOV + PRCV *PCV3				
	**Estimate**	**Std. Error**	***t*-value**	**Pr (>|t|)**
Intercept	−0.276	0.198	−1.424	0.1612
PCMV	0.297	0.148	2.005	0.0509
SOV	0.358	0.138	2.592	0.0128
PCV3	0.342	0.144	2.369	0.0221
Multiple R-squared	0.432	**F-statistic**	5.005	
Adjusted R-squared	0.346	***p*** **-value**	< 0.001	
Residual standard error	0.301			
**B. Individual results**				
Formula = PRCV ~ swIAV + PRRSV + PRRSV *swIAV + SOV + PCMV + PCV3 + PCV2 + PCMV *SOV + PCVM *PRRSV + SOV *PCV2				
	**Estimate**	**Std. Error**	***t*** **-value**	**Pr (**>**|t|)**
Intercept	0.531	0.280	1.932	0.059
PRRSV	−0.888	0.440	−2.016	0.049
Multiple *R^2^*	0.466	**F-statistic**	4.912	
Adjusted *R^2^*	0.491	***p*** **-value**	<0.001	
Residual standard error	0.382			
Formula = SOV ~ swIAV + PRRSV + PRRSV *swIAV + PCMV + PCV3 + PCV2 + PCMV *PRCV + PRCV *PRRSV				
	**Estimate**	**Std. Error**	***t*** **-value**	**Pr (**>**|t|)**
Intercept	0.658	0.164	3.990	<0.001
PCMV	−0.520	0.141	−3.679	<0.001
PCV2	0.474	0.167	2.829	0.006
Multiple *R^2^*	0.534	**F-statistic**	7.543	
Adjusted *R^2^*	0.464	***p*** **-value**	<0.001	
Residual standard error	0.369			
Formula = PCMV ~ swIAV + PRRSV + PRRSV *swIAV + SOV + PCV3 + PCV2 + SOV *PRCV + PRCV *PRRSV				
	**Estimate**	**Std. Error**	***t*** **-value**	**Pr (**>**|t|)**
Intercept	0.857	0.120	7.127	*p* < 0.001
PRCV	−0.332	0.124	−2.676	0.010
SOV	−0.454	0.122	−3.711	*p* < 0.001
Multiple *R^2^*	0.551	**F-statistic**	9.596	
Adjusted *R^2^*	0.493	***p-*** **value**	<0.001	
Residual standard error	0.350			

### Prevalence and association of pathogens on an individual level

**Figure 2:**
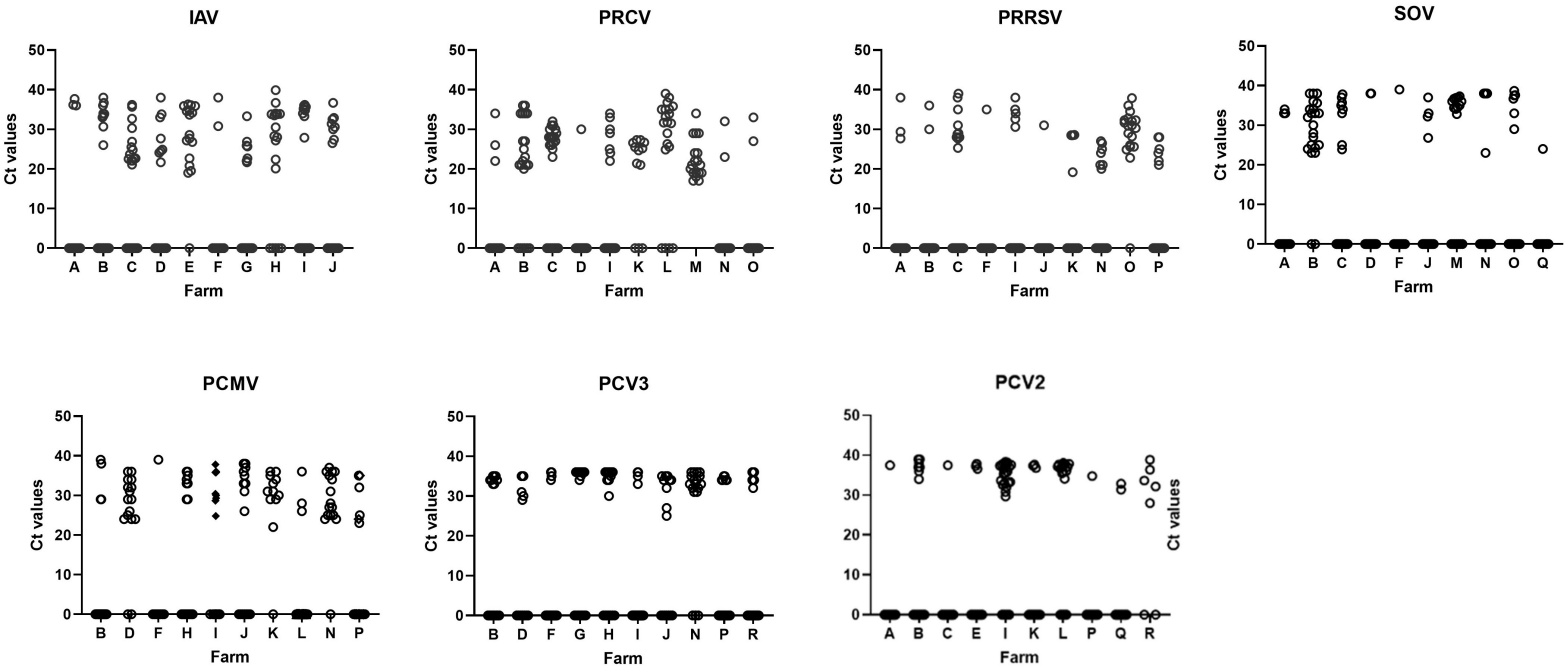
The figure shows the distribution of Ct values in 10 nurseries infected by each detected virus. To simplify the graphs, values for negative animals were shown as Ct = 0. The letter in the X-axis indicates the farm code. Detailed values of prevalence, confidence intervals and average Cts are shown in Supplementary material 1. IAV, influenza A virus; PRCV, porcine respiratory coronavirus; PRRSV, porcine reproductive and respiratory syndrome virus; SOV, swine orthopneumovirus; PCMV, porcine cytomegalovirus; PCV2, porcine circovirus 2; PCV3, porcine circovirus 3.

To have further insight on the prevalence of each pathogen, we then performed the analysis at the individual level. Ten batches (one batch of each farm) of infected nurseries for each pathogen were selected. As a result, a diversity of Cts (from 15–16 up to 30s) could be found for most of the examined viruses, while it was very uncommon to obtain Ct values of PCV2 and PCV3 below 30 (Figure 2; Supplementary material 2). On average, the individual prevalence for the different viruses were as follows: swIAV 48.6%; PRCV 48.0%; PRRSV 31.6%; SOV 33.8%; PCMV 48.3%, PCV2 36.0%; and PCV3 33.0% (Supplementary material 3). The presence of PCMV was negatively correlated with SOV and PRCV (*p* < 0.05) (Table 4B).

The correlation between different pathogens at the individual level was analyzed as well. As shown in Table 3, PRCV infection was negatively correlated with PRRSV and PCMV and positively associated with SOV (*p* < 0.05). PCMV was positively correlated with PRRSV (*p* < 0.05) and negatively correlated with SOV and PRCV (*p* < 0.05); SOV was positively correlated with PCV2 (*p* < 0.05). Apart from these, swIAV and PRRSV were negatively related (*p* < 0.05).

Next, the exploratory factorial analysis with 3 factors explained in total 55% of the variance (Supplementary material 1). Factor 1 comprised all variables but PCV3 (explaining 28% of the variance, loadings between −0.55 and 0.78). Factor 2 explained 15% of the variance and was composed of swIAV, PRCV, SOV, and PCMV (loadings between −0.64 and 0.62). Factor 3 explained 12% of the variance and was composed of PRCV and PCMV (loadings were −0.64 and 0.44, respectively).

The regression models for individual samples showed a negative correlation (*p* < 0.05) between PRCV and PRRSV. Presence of SOV in a sample was positively correlated with presence of PCV2 and negatively correlated with the presence of PCMV.

### Profiling of respiratory viruses in subclinically infected farms

**Figure 3:**
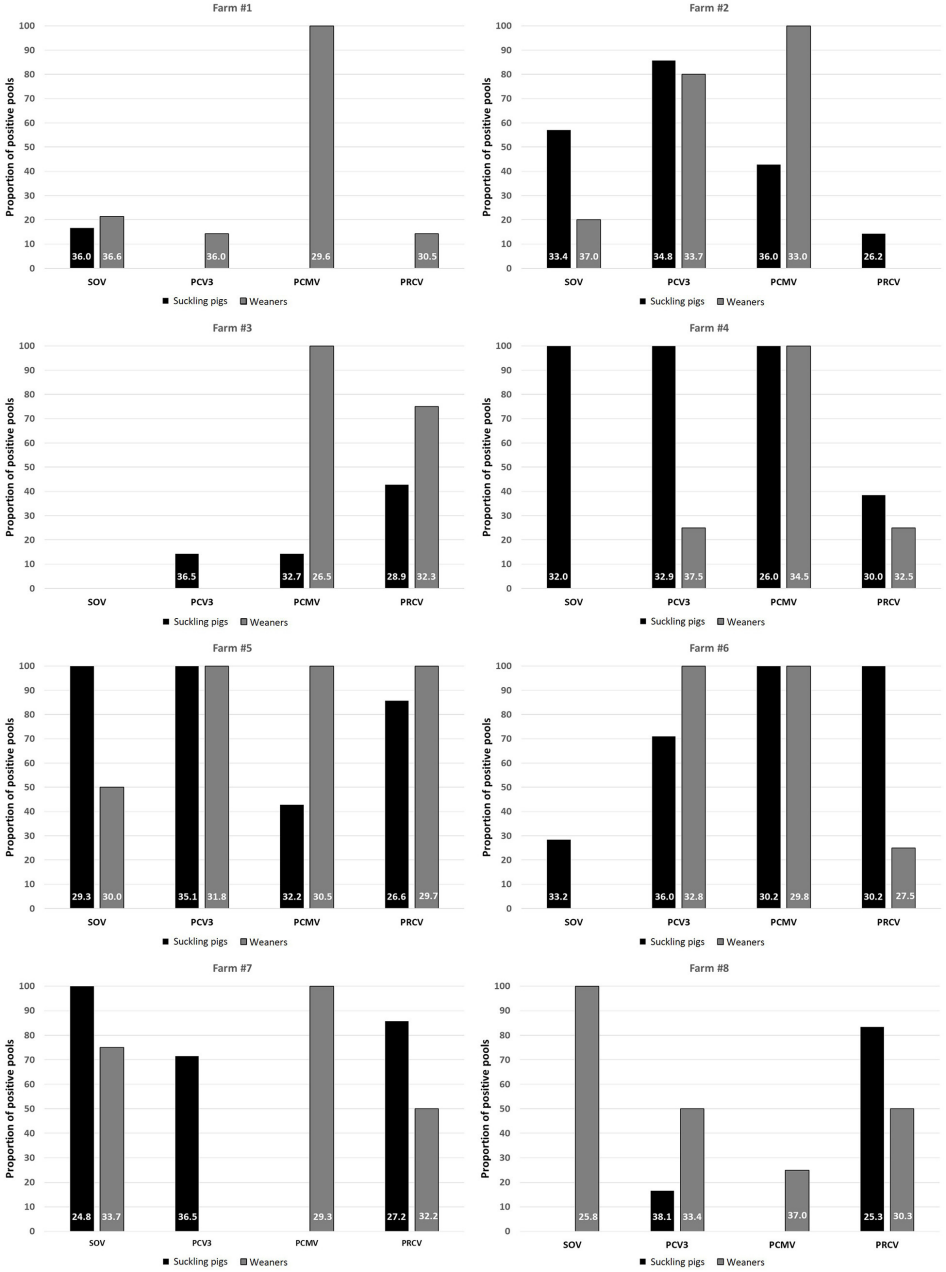
Proportion of positive pools for SOV, PCV3, PCMV and PRCV in suckling pigs and weaners among 8 monitored farms. The numbers within the bars indicate the average Ct value of the positive samples. PRCV, porcine respiratory coronavirus; SOV, swine orthopneumovirus; PCMV, porcine cytomegalovirus; PCV3, porcine circovirus 3.

To examine whether the infection by different viruses started from the maternal stage, 8 farms where suckling pigs and weaners had been sampled for influenza monitoring purposes were analyzed. In this part, only SOV, PCV3, PCMV, and PRCV were included because viral circulation of swIAV, PRRSV, and PCV2 may start in maternities. Analysis of nasal swabs indicated that PCMV spreads mainly in nurseries, since the frequency of positive pools in 7/8 farms reached 100% in that phase (Figure 3). In contrast, for SOV, PCV3, and PRCV, the infection was mostly found in suckling pigs, suggesting the role of sows in transmitting the infection. Interestingly in these subclinically infected farms, the tetrachoric correlation (Table 5) shows no significant correlation between examined viruses.

**Table 5:** Tetrachoric correlation matrix for results of nasal swabs collected in asymptomatic infected farms.

	**PRCV**	**SOV**	**PCV3**	**PCMV**
**Subclinical infection**
PRCV	1.00			
SOV	0.11	1.00		
PCV3	0.08	0.39	1.00	
PCMV	0.01	−0.07	0.09	1.00

The table shows the results of the tetrachoric correlation matrices for the presence or absence of each virus at farm with subclinical infection. All correlation values were non-significant (p > 0.05).

## Discussion

The polymicrobial nature of the porcine respiratory disease complex is a well-established and widely accepted concept. Nevertheless, the participation, contribution and interaction between different agents is not so well understood. One of the main obstacles is to establish experimental models that can reflect the agent complexity in a herd and the events taking place under farm conditions. Along with molecular technique advances, the number of viruses known to potentially participate in the respiratory disease in pigs has increased, with the discovery of IBV, IDV, PPIV1, SOV, PCV3, and PCV4 (20, 21, 23, 24, 27, 28, 43). New high-throughput methods such next generation sequencing, combined with novel detection methods as Fluidigm or Swinostics will increase the list of viruses that can be studied and improve the knowledge of their role in swine infectious diseases (44–46). However, the current cost of these instruments and techniques difficult their use in large scale studies. Real-time RT-PCR is sensitive enough and cost-effective.

In the present study, we examined the presence of a panel of 11 viruses in outbreaks of respiratory disease in nurseries. The cases included in this work had been initially submitted for swIAV diagnosis due to clinical signs of affected animals (cough and fever and increased mortality). Certainly, this would create a selection bias toward more severe cases of respiratory disease in nurseries. To compensate it, a comparable number of swIAV-positive and swIAV-negative outbreaks were balanced for analysis. Nasal swabs are the main sampling methods in the present study. Although nasal swabs are optimal for those viruses (i.e., swIAV, PRCV or PCMV) replicating in the nasal epithelium or higher airways, their use may underestimate other pathogens, such as PRRSV, whose replication in nasal mucosa may depend on viral virulence (47). This bias must be considered as well and, probably, favor the detection of more virulent isolates.

The analysis of pooled nasal swabs showed that IBV, IDV, PPIV1, and PCV4 were not present in any of the outbreaks analyzed, suggesting a low circulation, if not complete absence, of these pathogens in pig herds in Spain. Our results agree with a previous report (48).

Among 55 farms, up to 31 virus combinations were displayed, and interestingly, the presence of PRCV, SOV or PCMV was more frequently detected in swIAV positive nurseries. The cause for such association is unclear, it might reflect a poorer biosecurity in some farms, or the result of a confounding variable. The limited information available for those samples submitted for diagnosis makes virtually impossible to further investigate it.

At the individual level, the presence of swIAV in nasal swabs was negatively correlated with PRRSV shedding. *In vitro*, co-infection of susceptible epithelial CD163^+^ cells with PRRSV and swIAV interfered the replication of each other (49), while in pigs, some level of interference could also be observed when PRRSV infection preceded swIAV inoculation (50). The presence of PRRSV was found to be positively correlated with PCMV. In a previous study carried out in Chinese farms (51), PCMV positive animals were always found to be PRRSV-positive. Upregulation of IL-10 and downregulation of TNF-α and IL-8 by PCMV in porcine macrophages could be a reason for the interaction with PRRSV, but confirmation would require experimental efforts (15). Both PRRSV and PCMV were negatively correlated with PRCV and SOV. This observation is consistent with the known induction of type I interferon by PRCV (52) and the susceptibility of cytomegaloviruses to interferons (53–55).

Also, SOV and PRCV seemed to be positively related. Both agents were negatively correlated with PCMV, while SOV was positively related to PCV2. The fact that both PCMV and PCV2 are known immunosuppressive agents (56, 57), plus the ability of PRCV and other orthopneumoviruses for inducing interferon responses (58, 59), suggest a role of complex interactions of those viruses with the immune system. The confirmation of these associations would require either a different epidemiological study or, if possible, an experimental co-infection trial. To the best of our knowledge, no experimental infection with SOV has been reported.

Another interesting point to discuss is the different distribution of Ct values between most pathogens and PCV2 and PCV3 (Figure 2). While for swIAV, PRRSV, SOV, PCMV, nasal swabs in a farm most commonly contained either higher or lower viral loads (as deduced from Ct values), most animals yielded Ct values above 30 for PCV2 and PCV3. This suggests that shedding of PCV2 and PCV3 was relatively low. In the case of PCV2, this is reasonable because animals probably had high levels of maternally derived antibodies and were vaccinated at weaning. But for PCV3, the result is more difficult to explain. Zhai et al. (60) found a higher prevalence of PCV3 in weaners with severe respiratory disease indicating that Cts 20–27 were common in diseased animals, while Cts above 30 were found mostly in animals with less severe respiratory disease or asymptomatic. This could be the case of the present study.

Since for some farms we had samples from both suckling piglets and nurseries, we examined the presence of four of the tested viruses (SOV, PCV3, PRCV and PCMV) in both compartments within the same farm. The results revealed that all these agents started to circulate in the farrowing units, in a pattern already observed in other diseases. For example, transmission of swIAV in endemic farms has been well documented to start as early as the first week of age (61, 62). Interestingly, no correlation between the pathogens examined could be found when animals did not show evident signs of respiratory disease, indicating that serious respiratory disease is caused largely by co-infections.

In summary, the present study shows the complexity of the interaction between viruses present in nasal swabs of diseased, or subclinically infected animals, suggesting the existence of interactions among them that deserve further study. Moreover, we report the presence of SOV in Spain for the first time.

## Data availability statement

The raw data supporting the conclusions of this article will be made available by the authors, without undue reservation.

## Ethics statement

Ethical review and approval was not required for the animal study because the authors confirm that the ethical policies of the journal, as noted on the journal's author guidelines page have been adhered to. Samples used in this work were submitted by field veterinarians for diagnostic purposes on the basis of clinical testing. Therefore, according to Directive 63/2010 -Article 1, point 5(b) non-experimental clinical veterinary practices- the procedure carried out to obtain data is not within the scope of the named directive. Written informed consent for participation was not obtained from the owners because the data used in the present paper comes from routine diagnostic of pig farms. Also, the data in the present work is used anonymously.

## Author contributions

GM-V, YL, and EM participated in the design of the study. GM-V, YL, SS-P, ID, and EC performed the sample processing and analysis. EM performed the statistical analysis. All authors contributed in the writing process.

## Funding

This work was partially funded by CEVA Santé Animale (swIAV analysis).

## Conflict of interest

The authors declare that the research was conducted in the absence of any commercial or financial relationships that could be construed as a potential conflict of interest.

## Publisher's note

All claims expressed in this article are solely those of the authors and do not necessarily represent those of their affiliated organizations, or those of the publisher, the editors and the reviewers. Any product that may be evaluated in this article, or claim that may be made by its manufacturer, is not guaranteed or endorsed by the publisher.

## References

[1] Gillespie TG. Diagnosing endemic swine influenza virus in nursery pigs using crossectional serologic profiling. Swine Health Product. (1999) 7:81–3.

[2] Haimi-Hakala M Hälli O Laurila T Raunio-Saarnisto M Nokireki T Laine T. Etiology of acute respiratory disease in fattening pigs in Finland. Porc Health Manag. (2017) 3:19. 10.1186/s40813-017-0065-2 28852568 PMC5568250

[3] Qin S Ruan W Yue H Tang C Zhou K Zhang B. Viral communities associated with porcine respiratory disease complex in intensive commercial farms in Sichuan province, China. Sci Rep. (2018) 8:13341. 10.1038/s41598-018-31554-8 30190594 PMC6127300

[4] Ruggeri J Salogni C Giovannini S Vitale N Boniotti MB Corradi A. Association between infectious agents and lesions in post-weaned piglets and fattening heavy pigs with porcine respiratory disease complex (PRDC). Front Vet Sci. (2020) 7:636. 10.3389/fvets.2020.00636 33024748 PMC7516008

[5] Toya R Sasaki Y Uemura R Sueyoshi M. Indications and patterns of antimicrobial use in pig farms in the southern Kyushu, Japan: large amounts of tetracyclines used to treat respiratory disease in post-weaning and fattening pigs. J Vet Med Sci. (2021) 83:322–8. 10.1292/jvms.20-0436 33342965 PMC7972880

[6] Torremorell M Juarez A Chavez E Yescas J Doporto JM Gramer M. Procedures to eliminate H3N2 swine influenza virus from a pig herd. Vet Record. (2009) 165:74–7. 10.1136/vetrec.165.3.74 19617611

[7] O'Toole D Brown I Bridges A Cartwright SF. Pathogenicity of experimental infection with ‘pneumotropic' porcine coronavirus. Res Vet Sci. (1989) 47:23–9. 10.1016/S0034-5288(18)31226-8 2549594 PMC7125977

[8] Lanza I Brown IH Paton DJ. Pathogenicity of concurrent infection of pigs with porcine respiratory coronavirus and swine influenza virus. Res Vet Sci. (1992) 53:309–14. 10.1016/0034-5288(92)90131-K 1334565 PMC7131183

[9] Segalés J Domingo M Chianini F Majó N Domínguez J Darwich L. Immunosuppression in postweaning multisystemic wasting syndrome affected pigs. Vet Microbiol. (2004) 98:151–8. 10.1016/j.vetmic.2003.10.007 14741127

[10] Meng X-J. Porcine circovirus type 2 (PCV2): pathogenesis and interaction with the immune system. Ann Rev Anim Biosci. (2013) 1:43–64. 10.1146/annurev-animal-031412-103720 25387012

[11] Bordet E Blanc F Tiret M Crisci E Bouguyon E Renson P. Porcine reproductive and respiratory syndrome virus type 13 lena triggers conventional dendritic cells 1 activation and t helper 1 immune response without infecting dendritic cells. Front Immunol. (2018) 9:2299. 10.3389/fimmu.2018.02299 30333837 PMC6176214

[12] Nedumpun T Techakriengkrai N Thanawongnuwech R Suradhat S. Negative Immunomodulatory Effects of Type 2 Porcine Reproductive and Respiratory syndrome virus-induced interleukin-1 receptor antagonist on porcine innate and adaptive immune functions. Front Immunol. (2019) 10:579. 10.3389/fimmu.2019.00579 30972072 PMC6443931

[13] Li Y Mateu E. Interaction of type 1 porcine reproductive and respiratory syndrome virus with in vitro derived conventional dendritic cells. Front Immunol. (2021) 12:674185. 10.3389/fimmu.2021.674185 34177915 PMC8221110

[14] Liu X Xu Z Zhu L Liao S Guo W. Transcriptome analysis of porcine thymus following porcine cytomegalovirus infection. PLoS ONE. (2014) 9:e113921. 10.1371/journal.pone.0113921 25423176 PMC4244220

[15] Kavanová L Moutelíková R Prodělalová J Faldyna M Toman M Salát J. Monocyte derived macrophages as an appropriate model for porcine cytomegalovirus immunobiology studies. Vet Immunol Immunopathol. (2018) 197:58–62. 10.1016/j.vetimm.2018.01.008 29475507

[16] Opriessnig T Giménez-Lirola LG Halbur PG. Polymicrobial respiratory disease in pigs. Anim Health Res Rev. (2011) 12:133–48. 10.1017/S1466252311000120 22152290

[17] Lin X Huang C Shi J Wang R Sun X Liu X. Investigation of pathogenesis of H1N1 influenza virus and swine streptococcus suis serotype 2 co-infection in pigs by microarray analysis. PLoS ONE. (2015) 10:e0124086. 10.1371/journal.pone.0124086 25906258 PMC4407888

[18] Obradovic MR Segura M Segalés J Gottschalk M. Review of the speculative role of co-infections in Streptococcus suis-associated diseases in pigs. Vet Res. (2021) 52:49. 10.1186/s13567-021-00918-w 33743838 PMC7980725

[19] Saade G Deblanc C Bougon J Marois-Créhan C Fablet C Auray G. Coinfections and their molecular consequences in the porcine respiratory tract. Vet Res. (2020) 51:80. 10.1186/s13567-020-00807-8 32546263 PMC7296899

[20] Ducatez MF Pelletier C Meyer G. Influenza D virus in cattle, France, 2011-2014. Emerg Infect Dis. (2015) 21:368–71. 10.3201/eid2102.141449 25628038 PMC4313661

[21] Hause BM Ducatez M Collin EA Ran Z Liu R Sheng Z. Isolation of a novel swine influenza virus from Oklahoma in 2011 which is distantly related to human influenza C viruses. PLoS Pathog. (2013) 9:e1003176. 10.1371/journal.ppat.1003176 23408893 PMC3567177

[22] Ran Z Shen H Lang Y Kolb EA Turan N Zhu L. Domestic pigs are susceptible to infection with influenza B viruses. J Virol. (2015) 89:4818–26. 10.1128/JVI.00059-15 25673727 PMC4403465

[23] Tsai C-P Tsai H-J. Influenza B viruses in pigs, Taiwan. Influenza Other Respi Viruses. (2019) 13:91–105. 10.1111/irv.12588 29996007 PMC6304316

[24] Hause BM Padmanabhan A Pedersen K Gidlewski T. Feral swine virome is dominated by single-stranded DNA viruses and contains a novel orthopneumovirus which circulates both in feral and domestic swine. J Gen Virol. (2016) 97:2090–5. 10.1099/jgv.0.000554 27417702

[25] Dénes L Cságola A Schönhardt K Halas M Solymosi N Balka G. First report of porcine parainfluenza virus 1 (species Porcine respirovirus 1) in Europe. Transbound Emerg Dis. (2021) 68:1731–5. 10.1111/tbed.13869 33006252

[26] Ku J Wo A Cybulski P Denes L Balka G Stadejek T. Detection of porcine respirovirus 1 (PRV1) in Poland: incidence of co-infections with influenza A virus (IAV) and porcine reproductive and respiratory syndrome virus (PRRSV) in herds with a respiratory disease. Viruses. (2022) 14:148. 10.3390/v14010148 35062350 PMC8781826

[27] Lau SKP Woo PCY Wu Y Wong AYP Wong BHL Lau CCY. Identification and characterization of a novel paramyxovirus, porcine parainfluenza virus 1, from deceased pigs. J Gen Virol. (2013) 94:2184–90. 10.1099/vir.0.052985-0 23918408

[28] Chen S Zhang L Li X Niu G Ren L. Recent progress on epidemiology and pathobiology of porcine circovirus 3. Viruses. (2021) 13:1944. 10.3390/v13101944 34696373 PMC8538958

[29] Richard C-A Hervet C Ménard D Gutsche I Normand V Renois F. First demonstration of the circulation of a pneumovirus in French pigs by detection of anti-swine orthopneumovirus nucleoprotein antibodies. Vet Res. (2018) 49:118. 10.1186/s13567-018-0615-x 30518406 PMC6280484

[30] Park JY Welch MW Harmon KM Zhang J Piñeyro PE Li G. Detection, isolation, and in vitro characterization of porcine parainfluenza virus type 1 isolated from respiratory diagnostic specimens in swine. Vet Microbiol. (2019) 228:219–25. 10.1016/j.vetmic.2018.12.002 30593371

[31] Gaudino M Moreno A Snoeck CJ Zohari S Saegerman C O'Donovan T. Emerging Influenza D virus infection in European livestock as determined in serology studies: are we underestimating its spread over the continent? Transbound Emerg Dis. (2021) 68:1125–35. 10.1111/tbed.13812 32871031

[32] Sanogo IN Kouakou C Batawui K Djegui F Byarugaba DK Adjin R. Serological surveillance of influenza D virus in ruminants and swine in West and East Africa, 2017-2020. Viruses. (2021) 13:1749. 10.3390/v13091749 34578330 PMC8473344

[33] Schuele L Lizarazo-Forero E Cassidy H Strutzberg-Minder K Boehmer J Schuetze S. First detection of porcine respirovirus 1 in Germany and the Netherlands. Transbound Emerg Dis. (2021) 68:3120–5. 10.1111/tbed.14100 33837672 PMC9292642

[34] Zhang H Hu W Li J Liu T Zhou J Opriessnig T. Novel circovirus species identified in farmed pigs designated as porcine circovirus 4, Hunan province, China. Transbound Emerg Dis. (2020) 67:1057–61. 10.1111/tbed.13446 31823481

[35] Rakibuzzaman A Ramamoorthy S. Comparative immunopathogenesis and biology of recently discovered porcine circoviruses. Transbound Emerg Dis. (2021) 68:2957–68. 10.1111/tbed.14244 34288522

[36] Sosa Portugal S Cortey M Tello M Casanovas C Mesonero-Escuredo S Barrabés S. Diversity of influenza A viruses retrieved from respiratory disease outbreaks and subclinically infected herds in Spain (2017–2019). Transbound Emerg Dis. (2021) 68:519–30. 10.1111/tbed.13709 32619306 PMC8246522

[37] Schweiger B Zadow I Heckler R Timm H Pauli G. Application of a fluorogenic PCR assay for typing and subtyping of influenza viruses in respiratory samples. J Clin Microbiol. (2000) 38:1552–8. 10.1128/JCM.38.4.1552-1558.2000 10747142 PMC86487

[38] Olvera A Sibila M Calsamiglia M Segalés J Domingo M. Comparison of porcine circovirus type 2 load in serum quantified by a real time PCR in postweaning multisystemic wasting syndrome and porcine dermatitis and nephropathy syndrome naturally affected pigs. J Virol Methods. (2004) 117:75–80. 10.1016/j.jviromet.2003.12.007 15019262

[39] Busquets N Segalés J Córdoba L Mussá T Crisci E Martín-Valls GE. Experimental infection with H1N1 European swine influenza virus protects pigs from an infection with the 2009 pandemic H1N1 human influenza virus. Vet Res. (2010) 41:74. 10.1051/vetres/2010046 20663475 PMC2939699

[40] Faccini S de Mattia A Chiapponi C Barbieri I Boniotti MB Rosignoli C. Development and evaluation of a new Real-Time RT-PCR assay for detection of proposed influenza D virus. J Virol Methods. (2017) 243:31–4. 10.1016/j.jviromet.2017.01.019 28153610 PMC7113724

[41] Franzo G Legnardi M Centelleghe C Tucciarone CM Cecchinato M Cortey M. Development and validation of direct PCR and quantitative PCR assays for the rapid, sensitive, and economical detection of porcine circovirus 3. J Vet Diagn Invest. (2018) 30:538–44. 10.1177/1040638718770495 29629644 PMC6505917

[42] Sun W Du Q Han Z Bi J Lan T Wang W. Detection and genetic characterization of porcine circovirus 4 (PCV4) in Guangxi, China. Gene. (2021) 773:145384. 10.1016/j.gene.2020.145384 33383119

[43] Hou C-Y Zhang L-H Zhang Y-H Cui J-T Zhao L Zheng L-L. Phylogenetic analysis of porcine circovirus 4 in Henan Province of China: a retrospective study from 2011 to 2021. Transbound Emerg Dis. (2021) 69:1890–901. 10.1111/tbed.14172 34076964

[44] Cortey M Díaz I Vidal A Martín-Valls G Franzo G Gómez de Nova PJ. High levels of unreported intraspecific diversity among RNA viruses in faeces of neonatal piglets with diarrhoea. BMC Vet Res. (2019) 15:441. 10.1186/s12917-019-2204-2 31805938 PMC6896758

[45] Goecke NB Hjulsager CK Krog JS Skovgaard K Larsen LE. Development of a high-throughput real-time PCR system for detection of enzootic pathogens in pigs. J Vet Diagn Invest. (2020) 32:51–64. 10.1177/1040638719890863 31752620 PMC7003221

[46] Montagnese C Barattini P Giusti A Balka G Bruno U Bossis I. A diagnostic device for in-situ detection of swine viral diseases: the swinostics project. Sensors. (2019) 19:407. 10.3390/s19020407 30669504 PMC6359211

[47] Frydas IS Nauwynck HJ. Replication characteristics of eight virulent and two attenuated genotype 1 and 2 porcine reproductive and respiratory syndrome virus (PRRSV) strains in nasal mucosa explants. Vet Microbiol. (2016) 182:156–62. 10.1016/j.vetmic.2015.11.016 26711043

[48] Franzo G Ruiz A Grassi L Sibila M Drigo M Segalés J. Lack of porcine circovirus 4 Genome Detection in Pig Samples from Italy and Spain. Pathogens. (2020) 9:433. 10.3390/pathogens9060433 32486429 PMC7350368

[49] Provost C Hamonic G Gagnon CA Meurens F. Dual infections of CD163 expressing NPTr epithelial cells with influenza A virus and PRRSV. Vet Microbiol. (2017) 207:143–8. 10.1016/j.vetmic.2017.06.012 28757015

[50] Dobrescu I Levast B Lai K Delgado-Ortega M Walker S Banman S. In vitro and ex vivo analyses of co-infections with swine influenza and porcine reproductive and respiratory syndrome viruses. Vet Microbiol. (2014) 169:18–32. 10.1016/j.vetmic.2013.11.037 24418046 PMC7117334

[51] Liu X Liao S Zhu L Xu Z Zhou Y. Molecular epidemiology of porcine cytomegalovirus (PCMV) in Sichuan Province, China: 2010-2012. PLoS ONE. (2013) 8:e0064648. 10.1371/journal.pone.0064648 23762243 PMC3675093

[52] van Reeth K Labarque G Nauwynck H Pensaert M. Differential production of proinflammatory cytokines in the pig lung during different respiratory virus infections: correlations with pathogenicity. Res Vet Sci. (1999) 67:47–52. 10.1053/rvsc.1998.0277 10425240 PMC7126504

[53] Buddaert W van Reeth K Pensaert M. In vivo and in vitro interferon (IFN) studies with the porcine reproductive and respiratory syndrome virus (PRRSV). Adv Exp Med Biol. (1998) 440:461–7. 10.1007/978-1-4615-5331-1_59 9782316

[54] Lee S-M Schommer SK Kleiboeker SB. Porcine reproductive and respiratory syndrome virus field isolates differ in in vitro interferon phenotypes. Vet Immunol Immunopathol. (2004) 102:217–31. 10.1016/j.vetimm.2004.09.009 15507307 PMC7112598

[55] Holzki JK Dag F Dekhtiarenko I Rand U Casalegno-Garduño R Trittel S. Type I interferon released by myeloid dendritic cells reversibly impairs cytomegalovirus replication by inhibiting immediate early gene expression. J Virol. (2015) 89:9886–95. 10.1128/JVI.01459-15 26202227 PMC4577895

[56] Darwich L Mateu E. Immunology of porcine circovirus type 2 (PCV2). Virus Res. (2012) 164:61–7. 10.1016/j.virusres.2011.12.003 22178803

[57] Dix RD Mocarski E Rowland-Jones S Raj A Patro K. Subversion of Immune Response by Human Cytomegalovirus. Front Immunol. (2019) 1:1155. 10.3389/fimmu.2019.01155 31244824 PMC6575140

[58] Russell CD Unger SA Walton M Schwarze J. The human immune response to respiratory syncytial virus infection. Clin Microbiol Rev. (2017) 30:481–502. 10.1128/CMR.00090-16 28179378 PMC5355638

[59] Saif LJ Jung K. Comparative pathogenesis of bovine and porcine respiratory coronaviruses in the animal host species and SARS-CoV-2 in humans. J Clin Microbiol. (2020) 58:e01355-20. 10.1128/JCM.01355-20 32522830 PMC7383540

[60] Zhai S-L Zhou X Zhang H Hause BM Lin T Liu R. Comparative epidemiology of porcine circovirus type 3 in pigs with different clinical presentations. Virol J. (2017) 14:222. 10.1186/s12985-017-0892-4 29132394 PMC5683367

[61] Bhatta TR Ryt-Hansen P Nielsen JP Larsen LE Larsen I Chamings A. Infection dynamics of swine influenza virus in a danish pig herd reveals recurrent infections with different variants of the H1N2 swine influenza A virus subtype. Viruses. (2020) 12:1013. 10.3390/v12091013 32927910 PMC7551734

[62] Garrido-Mantilla J Sanhueza J Alvarez J Culhane MR Davies P Allerson MW. Impact of nurse sows on influenza A virus transmission in pigs under field conditions. Prev Vet Med. (2021) 188:105257. 10.1016/j.prevetmed.2021.105257 33472145

