# Supplementary material for: Diversity of respiratory viruses present in nasal swabs under influenza suspicion in respiratory disease cases of weaned pigs
Source: Front Vet Sci. 2022 Oct 19;9:1014475. doi: 10.3389/fvets.2022.1014475 (PMC9627340; doi:10.3389/fvets.2022.1014475)
Supplement: Supplementary file 1 [file Data_Sheet_1.docx]

**Supplementary material 1. Results of the exploratory factorial analysis.**

1. **Farm level**

|  | **swIAV** | **PRRSV** | **PRCV** | **SOV** | **PCV2** | **PCV3** | **PCMV** | **Overall** |
| --- | --- | --- | --- | --- | --- | --- | --- | --- |
| **KMO-MSA*** | 0.79 | 0.54 | 0.67 | 0.71 | 0.77 | 0.48 | 0.75 | 0.69 |
| **Bartlett test** |  |  |  |  |  |  |  | Χ^2^=53.34  p=0.0001 |
| **Uniqueness** | 0.765 | 0.970 | 0.420 | 0.544 | 0.928 | 0.005 | 0.555 |  |
| **Eigenvalues**  **(7 components)** | 2.39; 1.15; 0.95; 0.87; 0.67; 0.57; 0.39 | | | | | | | |

*KMO = Kaiser-Meyer-Olkin test

|  | **Loadings** | |
| --- | --- | --- |
| **Variable** | **Factor 1** | **Factor 2** |
| **swIAV** | 0.426 | 0.232 |
| **PRRSV** | 0.159 |  |
| **PRCV** | 0.689 | 0.325 |
| **SOV** | 0.674 |  |
| **PCV2** | 0.212 | 0.164 |
| **PCV3** |  | 0.997 |
| **PCMV** | 0.663 |  |
| **SS loadings** | 1.619 | 1.119 |
| **% variance** | 0.231 | 0.170 |
| **Cumulative variance** | 0.231 | 0.402 |
| **Χ^2/^p-value** | 1.87/p=0.985 | |

1. Individual level

|  | **swIAV** | **PRRSV** | **PRCV** | **SOV** | **PCV2** | **PCV3** | **PCMV** | **Overall** |
| --- | --- | --- | --- | --- | --- | --- | --- | --- |
| **KMO-MSA*** | 0.61 | 0.61 | 0.73 | 0.68 | 0.53 | 0.73 | 0.70 | 0.68 |
| **Bartlett test** |  |  |  |  |  |  |  | Χ2=13.61  p=0.030 |
| **Uniqueness** | 0.00 | 0.77 | 0.33 | 0.00 | 0.78 | 0.92 | 0.38 |  |
| **Eigenvalues**  **(7 components)** | 2.60; 1.11; 1.010; 0.95; 0.58; 0.39; 0.29 | | | | | | | |

|  | **Loadings** | | |
| --- | --- | --- | --- |
| **Variable** | **Factor 1** | **Factor 2** | **Factor 3** |
| **swIAV** | 0.77 | -0.64 |  |
| **PRRSV** | 0.36 |  |  |
| **PRCV** | 0.39 | 0.33 | -0.64 |
| **SOV** | 0.78 | 0.62 |  |
| **PCV2** | 0.39 |  |  |
| **PCV3** |  |  |  |
| **PCMV** | -0.55 | -0.35 | 0.44 |
| **SS loadings** | 1.94 | 1.06 | 0.8 |
| **% variance** | 0.28 | 0.15 | 0.12 |
| **Cumulative variance** | 0.28 | 0.43 | 0.55 |
| **Χ^2/^p-value** | 1.92/p-value = 0.589 | | |

**Supplementary material 2. Distribution of Ct values at individual level.** The box and whiskers graph show the distribution of Ct values for each of the examined pathogens. The values shown are: minimum, quartile 25%, mean, quartile 75%, maximum.

**Supplementary material 3. Detail of the prevalence, confidence intervals and average Ct value for each pathogen as resulting from the analysis of the individual nasal swabs by PCR.**

1. **swIAV**

| **Farm** | **Proportion of positive samples**  **(95% confidence Interval)** | **Mean Ct** |
| --- | --- | --- |
| **A** | 25.0% (CI_95%_: 6.7-57.2%) | 36.3 |
| **B** | 40.9% (CI_95%_: 21.5-64.4%) | 33.5 |
| **C** | 60.9% (CI_95%_: 38.8-79.5%) | 26.4 |
| **D** | 50.0% (CI_95%_: 25.5-74.5%) | 28.4 |
| **E** | 94.1% (CI_95%_: 69.2-99.7%) | 29.6 |
| **F** | 22.2% (CI_95%_: 4.0-59.8%) | 34.4 |
| **G** | 35.0% (CI_95%_: 16.3-59.1%) | 25.5 |
| **H** | 72.2% (CI_95%_: 46.4-89.3%) | 30.9 |
| **I** | 36.0% (CI_95%_: 18.7-57.4%) | 34.1 |
| **J** | 50.0% (CI_95%_: 25.5-74.5%) | 31.0 |
| **Average** | **48.6%** | **31.0** |

**b) PRCV**

| **Farm** | **Proportion of positive samples**  **(95% confidence Interval)** | **Mean Ct** |
| --- | --- | --- |
| **A** | 25.0% (CI_95%_: 6.7-57.2%) | 27.3 |
| **B** | 72.7% (CI_95%_: 49.6-88.7%) | 27.2 |
| **C** | 69.6% (CI_95%_: 49.6-85.9%) | 28.0 |
| **D** | 6.3% (CI_95%_: 0.3-32.3%) | 30.0 |
| **I** | 75.0% (CI_95%_: 42.8-93.3%) | 25.1 |
| **K** | 80.0% (CI_95%_: 55.7-93.4%) | 32.7 |
| **L** | 100% (CI_95%_: 78.2-100%) | 22.4 |
| **M** | 13.3% (CI_95%_: 2.3-41.6%) | 30.9 |
| **N** | 28.0% (CI_95%_: 12.9-49.6%) | 28.1 |
| **O** | 10.5% (CI_95%_: 1.8-39.5%) | 30.0 |
| **Average** | **48.0%** | **28.2** |

**c) PRRSV**

| **Farm** | **Proportion of positive samples**  **(95% confidence Interval)** | **Mean Ct** |
| --- | --- | --- |
| **A** | 25.0% (CI_95%_: 6.7-57.2%) | 31.7 |
| **B** | 9.1% (CI_95%_: 1.6-30.6%) | 33.0 |
| **C** | 43.5% (CI_95%_: 23.9-65.1%) | 31.0 |
| **F** | 33.3% (CI_95%_: 11.3-64.6%) | 26.2 |
| **I** | 10.0% (CI_95%_: 0.5-45.9%) | 35.0 |
| **J** | 41.2% (CI_95%_: 19.4-66.6%) | 23.5 |
| **K** | 33.3% (CI_95%_: 14.4-58.6%) | 24.7 |
| **N** | 20.0% (CI_95%_: 7.6-41.3%) | 30.9 |
| **O** | 6.3% (CI_95%_: 0.3-32.3%) | 31.0 |
| **P** | 94.7% (CI_95%_: 71.9-99.7%) | 30.0 |
| **Average** | **31.6%** | **29.7** |

**d) SOV**

| **Farm** | **Proportion of positive samples**  **(95% confidence Interval)** | **Mean Ct** |
| --- | --- | --- |
| **A** | 25.0% (CI_95%_: 6.7-57.2%) | 33.3 |
| **B** | 95.5% (CI_95%_: 75.1-99.8%) | 30.7 |
| **C** | 36.4% (CI_95%_: 18.0-59.2%) | 32.7 |
| **D** | 12.5% (CI_95%_: 2.2-39.5%) | 38.0 |
| **F** | 10.0% (CI_95%_: 0.5-45.9%) | 39.0 |
| **J** | 25.0% (CI_95%_: 8.3-52.6%) | 32.3 |
| **M** | 61.1% (CI_95%_: 36.1-81.7%) | 35.5 |
| **N** | 31.3% (CI_95%_: 12.1-58.5%) | 35.0 |
| **O** | 31.6% (CI_95%_: 13.6-56.5%) | 35.4 |
| **Q** | 10.0% (CI_95%_: 0.5-45.9%) | 32.4 |
| **Average** | **33.8%** | **34.4** |

**e) PCMV**

| **Farm** | **Proportion of positive samples**  **(95% confidence Interval)** | **Mean Ct** |
| --- | --- | --- |
| **B** | 18.2% (CI_95%_: 6.0-41.0%) | 33.8 |
| **D** | 87.5% (CI_95%_: 60.4-97.8%) | 29.7 |
| **F** | 10.0% (CI_95%_: 0.5-45.9%) | 39.0 |
| **H** | 44.4% (CI_95%_: 22.4-68.7%) | 33.1 |
| **I** | 36.0% (CI_95%_: 18.7-57.4%) | 32.1 |
| **J** | 56.3% (CI_95%_: 30.6-79.3%) | 34.1 |
| **K** | 91.7% (CI_95%_: 59.8-99.6%) | 31.5 |
| **L** | 12.0% (CI_95%_: 3.2-32.3%) | 30.0 |
| **N** | 93.8% (CI_95%_: 67.7-99.7%) | 30.0 |
| **P** | 33.3% (CI_95%_: 14.4-58.9%) | 29.0 |
| **Average** | **48.3%** | **32.2** |

**f) PCV2**

| **Farm** | **Proportion of positive samples**  **(95% confidence Interval)** | **Mean Ct** |
| --- | --- | --- |
| **A** | 8.3% (CI_95%_: 0.4-40.3%) | 37.5 |
| **B** | 31.8% (CI_95%_: 14.7-54.9%) | 37.1 |
| **C** | 4.4% (CI_95%_: 0.2-24.0%) | 37.5 |
| **E** | 17.7% (CI_95%_: 4.7-44.2%) | 37.3 |
| **I** | 80.8% (CI_95%_: 60.0-92.7%) | 35.3 |
| **K** | 25.0% (CI_95%_: 6.7-57.2%) | 37.3 |
| **L** | 70.0% (CI_95%_: 45.7-87.2%) | 36.8 |
| **P** | 12.5% (CI_95%_: 2.2-39.6%) | 32.2 |
| **Q** | 5.6% (CI_95%_: 0.3-29.4%) | 34.8 |
| **R** | 71.4% (CI_95%_: 30.3-94.9%) | 33.8 |
| **Average** | **32.8%** | **36.0** |

**g) PCV3**

| **Farm** | **Proportion of positive samples**  **(95% confidence Interval)** | **Mean Ct** |
| --- | --- | --- |
| **B** | 47.1% (CI_95%_: 23.9-71.5%) | 34.1 |
| **D** | 41.7% (CI_95%_: 16.5-71.4%) | 32.0 |
| **F** | 20.0% (CI_95%_: 6.6-44.3%) | 32.7 |
| **G** | 50.0% (CI_95%_: 26.8-73.2%) | 34.5 |
| **H** | 56.3% (CI_95%_: 30.6-79.3%) | 34.7 |
| **I** | 16.0% (CI_95%_: 5.3-36.9%) | 35.0 |
| **J** | 56.3% (CI_95%_: 30.6-79.3%) | 33.3 |
| **N** | 83.3% (CI_95%_: 57.7-95.6%) | 33.8 |
| **P** | 33.3% (CI_95%_: 14.4-38.9%) | 34.3 |
| **R** | 35.0% (CI_95%_: 16.3-59.5%) | 25.3 |
| **Average** | **43.9%** | **33.0** |
